# Whole-genome sequencing identifies nosocomial transmission of extra-pulmonary *M. tuberculosis*

**DOI:** 10.1093/qjmed/hcw202

**Published:** 2016-12-22

**Authors:** T.M. Walker, D.W. Crook, T.E.A. Peto, C.P. Conlon

**Affiliations:** From the 1 Oxford University Hospitals NHS Trust, Oxford, UK; 2 Nuffield Department of Medicine, University of Oxford; 3 NIHR Oxford Biomedical Research Centre, John Radcliffe Hospital, Oxford, UK

Learning point for clinicians

- Hospital infection control practice reflects the working assumption that extra pulmonary tuberculosis is not considered infectious. We describe how whole-genome sequencing identified the nosocomial transmission of extra-pulmonary *Mycobacterium tuberculosis* infection on our Infectious Diseases unit in Oxford, UK.
- Suppurating wounds in tuberculosis constitute a hazard requiring risk assessment for transmission. We have re-consideration our infection control practices in our hospital in the light of this case to account for the potential of tuberculosis transmission in settings other than just pulmonary disease. We recommend others consider similar measures.

## Case report

Infection control practices in clinical areas are designed to prevent *Mycobacterium tuberculosis* being transmitted via airway emitted droplet nuclei.^1^ According to this paradigm pulmonary smear positive disease is considered more infectious than smear negative disease,^2^ whereas extra-pulmonary disease, which should not generate droplet nuclei, is not considered infectious. To monitor tuberculosis transmission in Oxfordshire, UK, we have been prospectively whole-genome sequencing (WGS) all clinical *M. tuberculosis* isolates since 2011, and retrospectively to 2007.^3^ Here we describe a case of nosocomial transmission of extra-pulmonary tuberculosis identified in the process.

## Case1

**Figure 1:**
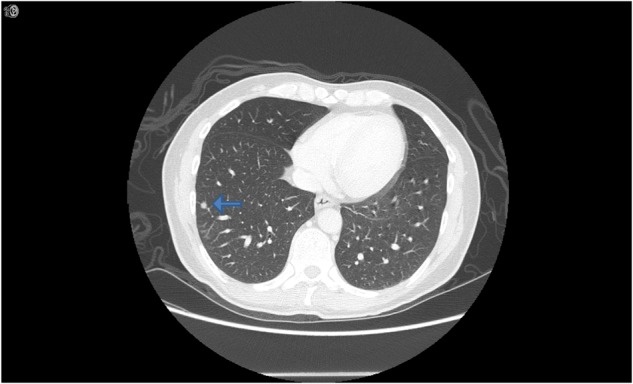
CT thorax image of case 1. Nodule seen in peripheral right lung field.

A 46-year-old UK-born patient presented to our infectious diseases unit with fever and 3 × 3 cm axillary and inguinal lymphadenopathy. The patient had recently been started on anti-retroviral (ARV) therapy for HIV infection with a CD4 lymphocyte count of 190 cells/mm^3^ and a viral load of over 500 000 copies/ml. CT imaging of the thorax and abdomen demonstrated extensive lymphadenopathy, but only two small pulmonary nodules in the upper lobes (Figure 1). There was no cough. Microscopy of an axillary lymph node biopsy identified acid-fast bacilli with *M. tuberculosis* infection confirmed on culture and the patient was commenced on standard quadruple anti-tuberculosis therapy. The patient suffered an immune reconstitution like-syndrome with suppurating lymph nodes requiring daily dressing whilst remaining culture positive for 3 weeks. The patient eventually made a good response to both tuberculosis therapy, and to ARVs.

## Case 2

A 41-year-old South Asian-born nurse presented 9 months later with a 2-week history of fever, abdominal pain, and 48 h of vomiting. Abdominal CT imaging revealed peritoneal nodularity and ascites with laparoscopic findings characteristic of tuberculosis infection, confirmed on culture. HIV testing was negative and the patient successfully responded to standard tuberculosis chemotherapy.

## Strain typing and epidemiology

Both patients’ isolates underwent routine 24-locus MIRU-VNTR (mycobacterial interspersed repetitive unit-variable number tandem repeat) typing, producing identical profiles. The isolates later underwent WGS using Illumina platforms, as described previously.^3^ Zero single nucleotide polymorphisms (SNPs) separated the two patient samples with no other sequenced *M. tuberculosis* cultures from Oxfordshire since 2007 within 12 SNPs, making direct transmission from a third case highly unlikely.^4^

As MIRU-VNTR lacks specificity,^4^ the matching results did not initially lead to an investigation as neither patient was regarded infectious, and as they associated with different social circles in separate towns. The genomic link was therefore a surprise, and we investigated possible explanations: Although both patients might have been infected by a third party whose isolate had not been sequenced, there were no other samples from Oxfordshire with a matching MIRU-VNTR profile either. Laboratory contamination was thought unlikely as the samples were obtained 9 months apart. After reviewing the first patient’s journey in more detail, we realised the second patient had nursed them during their hospital stay, regularly dressing the suppurating lymph nodes. We surmise that this process aerosolised tubercle bacilli that were inhaled or ingested by the second patient (the nurse).

## Conclusion

The specificity of WGS data indicated a high prior probability that transmission between these two apparently non-infectious individuals had occurred,^4^ leading to a reconsideration of the mode of transmission between them. Although the infection of laboratory workers from handling clinical specimens or cultures has been described,^4^ to the best of our knowledge there has not been a recorded case of nosocomial, person-to-person transmission of extra-pulmonary tuberculosis.

## References

[1] WHO Policy on TB Infection Control in Health-Care Facilities, Congregate Settings and Households. Geneva, World Health Organization, 2009. 24432438

[2] Guerra-Assunção JA Crampin AC Houben R Mzembe T Mallard K Coll F, Large-scale whole genome sequencing of M. tuberculosis provides insights into transmission in a high prevalence area. Karim QA, editor. eLife 2015; 4: e05166. 10.7554/eLife.05166 PMC4384740 25732036

[3] Walker TM Lalor MK Broda A Saldana Ortega L Morgan M Parker L, Assessment of Mycobacterium tuberculosis transmission in Oxfordshire, UK, 2007-12, with whole pathogen genome sequences: an observational study. Lancet Respir Med 2014; 2:285–92. 24717625 10.1016/S2213-2600(14)70027-X PMC4571080

[4] Walker TM Ip CL Harrell RH Evans JT Kapatai G Dedicoat MJ, Whole-genome sequencing to delineate Mycobacterium tuberculosis outbreaks: a retrospective observational study. Lancet Infect Dis 2013; 13:137–46. 23158499 10.1016/S1473-3099(12)70277-3 PMC3556524

